# Project SUMS (scaling up of mental health in schools): design and methods for a pragmatic, cluster randomised waitlist-controlled trial on integrated school mental health intervention for adolescents

**DOI:** 10.1186/s12889-021-12086-9

**Published:** 2021-11-06

**Authors:** Senthil Amudhan, Kavita Jangam, Kalaivani Mani, Nithya Poornima Murugappan, Eesha Sharma, Payel Mahapatra, Ajit Deo Burma, Hemant Kumar Tiwari, Ashi Ashok, Sangappa Vaggar, Girish Nagaraja Rao

**Affiliations:** 1 Department of Epidemiology, National Institute of Mental Health And Neuro Sciences, Bangalore, Karnataka India; 2 Department of Psychiatric Social Work, National Institute of Mental Health And Neuro Sciences, Bangalore, Karnataka India; 3 Department of Biostatistics, All India Institute of Medical Sciences, New Delhi, Delhi India; 4 Clinical Psychologist, Independent Consultant, Bangalore, Karnataka India; 5 Department of Child and Adolescent Psychiatry, National Institute of Mental Health And Neuro Sciences, Bangalore, Karnataka India

**Keywords:** Mental health literacy, Mental health promotion, First aid in mental health, School mental health, Resilience, Cluster randomised trial

## Abstract

**Background:**

There is an increasing need for Mental Health Promotion (MHP) among adolescents, especially in developing countries with limited resources and rapid socio-demographic transition. With the growing burden of mental health problems among adolescents (suicide, depression) and their preferences to seek help from their peers, improving Mental Health Literacy (MHL) and behaviours for First Aid in Mental Health (MH-FA) becomes crucial to promote their mental health.

**Methods:**

Schools are ideal settings for reaching the vulnerable adolescents. The proposed study evaluates the effectiveness of a classroom-based teacher-led integrated school mental health intervention called SUMS (MHP + MHL + MH-FA). The study will involve a pragmatic, cluster-randomised waitlist-controlled design to evaluate the effectiveness of SUMS intervention using schools as unit-of-randomisation. The study will be conducted in Srinivaspura taluka (Sub-district) of Kolar district (administrative unit of health) of Karnataka in collaboration with a multi-disciplinary expert team from NIMHANS (National Institute of Mental Health And Neuro Sciences), Bangalore-India and Department of Education, Government of Karnataka, India. A total of 8 schools (400 students studying in 6–8 grade) from Srinivaspura taluka will be randomised into intervention and waitlist control group. The intervention group will receive SUMS intervention through 10–15 h of classroom sessions. The primary outcome is the improvement in positive mental health literacy, as measured by the Mental Health-Promoting Knowledge (MHPK-10) scale. Changes in MH-FA knowledge and intentions, Mental health stigma, help-seeking and resilience are assessed as secondary outcomes. Data will be collected at baseline, 6-weeks, 6-months and 12-months post-intervention. The waitlist-control schools will receive the interventions at the end of the 12-month follow-up assessment in intervention-schools.

**Discussion:**

This is the first study to integrate Mental Health Literacy with Mental Health Promotion and behaviours for First Aid in Mental Health to promote mental health well-being among adolescent school children in India. With a need to build a more substantial evidence base on School Mental Health Promotion approaches in developing countries, the study findings will have implications for implementing and operationalising Health and Wellness Ambassador initiative in India.

**Trial registration:**

Clinical Trials Registry - India, CTRI/2019/07/020394. Registered prospectively on 29 July 2019. (ctri.nic.in/Clinicaltrials/pmaindet2.php?trialid=35724&EncHid=&userName=sums).

## Background

In recent years, there is an increasing concern about adolescents’ mental health, especially in developing countries. An estimated 10–20% of children and adolescents are affected by mental health problems worldwide [1]. Adolescence is a period that is characterised by rapid physical and psychological changes with altered social perceptions and expectations [2]. The expectations and the choices made during this period have a significant impact not only on current health-practices and well-being but also on their health as adults. We must engage with them and enable them to make right choices about their expectations and address their mental health needs effectively through mental health promotion (MHP). With rapid socio-demographic and lifestyle transition, such mental health promotion will help adolescents build resilience to cope well in difficult situations or adversities. This will be critical not only for their well-being during adolescence but also for their physical and mental health in adulthood.

Today, suicide is one of the leading cause of preventable deaths, and depression is one of the leading cause of morbidity among adolescents [3]. Based on the life-course perspective, it has been emphasised to target adolescents in school to accommodate the contextual factors that influence suicide during adolescence [4]. Even though these mental health problems have detrimental effects on well-being, and development in adolescence, a reluctance to seek professional help due to stigma and lack of mental health knowledge has been documented in various studies across the globe [5–7]. Thus, there is a need to improve help-seeking to reduce the growing burden of mental health problems among adolescents and promote mental health literacy as an important strategy to promote help-seeking among adolescents [8–10].

Mental health literacy (MHL) is defined as “knowledge and attitudes regarding mental health that aid in recognition, management and prevention of mental health issues” [11]. Low levels of MHL were associated with depression and MHL among adolescents was positively associated with their mental health [12, 13]. Even though mental health literacy has significant implications on adolescent mental health, poor mental health literacy has been demonstrated for adolescents across various settings [14, 15]. Thus, MHL must become a focus of mental health interventions for adolescents. With the adolescents preferring to disclose mental illness symptoms to their peers and reluctant to seek professional help, the importance of improving behaviours for First Aid in Mental Health (MH-FA) to facilitate social support and help-seeking for mental illness cannot be overlooked. Mental health first aid is the help provided to a person who is developing a mental health problem, experiencing a worsening of an existing mental health problem or in a mental health crisis [16]. As adolescents begin to make decisions about their health and often have a preference for seeking help from peers, mental health literacy to adolescents should be provided ideally as a part of the school curriculum and not as ad-hoc events that occur infrequently (such as mental health parades, campaigns etc.,) [8]. It is further emphasised that schools should promote not only positive mental health but also MHL to enable students to understand mental health problems, reduce mental health stigma, promote mental health self-care and help-seeking behaviours of students [13]. Thus, integration of First Aid in Mental Health (MH-FA) with MHP and MHL, becomes a potential strategy for mental health promotion and prevention in adolescents.

Given the critical role of education in health literacy, schools become an ideal setting for reaching vulnerable adolescents for improving mental health literacy. This is because adolescents spend most of their time in schools, and health is intrinsically linked with education. Furthermore, many risks and protective factors for adverse mental health outcomes are operated in the school context. Presently, the evidence base for educational interventions targeting adolescents for mental health literacy and mental health promotion is limited [17]. Even though there is a concept of Health Promoting Schools (HPS) by World Health Organization that provides a broad framework for action, there is little evidence on schools’ ability to implement this approach. Existing evaluations of schools that had implemented HPS initiative have shown that mental health (despite being an important outcome of HPS initiative) was not addressed, apart from substance use [18, 19]. A clear need for well-controlled research in the area of mental health literacy and mental health promotion has been documented due to previous studies with small sample sizes, lack of follow-up, low response rates and potential contamination [20].

In India, adolescent mental health assumes a great relevance where adolescents aged 10–19 years constitute about 22% of the population [21]. With increasing enrolment in secondary schools in India, schools become the ideal platform for promoting positive mental health among adolescents. Even though there are Adolescence Education Programme and Rashtriya Kishor Swasthya Karyakram programme in India, it was evident that such programmes have either failed to account for the mental health needs of school communities or failed to measure the changes or values that are intended for the sustenance of mental health promotion [22, 23].

Developing personal skills for both students and teachers has been emphasised for successful mental health interventions [13] Among various approaches to address mental health literacy in schools, a natural approach builds on schools’ existing social ecologies such as curriculum presented by usual classroom teachers. Also, teacher-led approaches were found to facilitate the creation of a school-wide environment of acceptance and normalisation of mental illnesses as well as facilitate recognition and help-seeking in adolescents [8]. MHL delivered by external providers had limited value on the sustained and enhanced MHL capacity embedded into educational systems [13]. Further, MHL delivered by teachers was found to have positive mental health literacy outcomes across various settings and teacher delivered MHL curriculum found to have an advantage in normalising mental health knowledge as a part of education [13].

With this in the background, we developed a classroom-based teacher-led integrated school mental health intervention called SUMS (MHP + MHL + MH-FA) based on “School Friendly” mental health literacy approach and “School-Based Integrated Pathway to Care Model” developed by Kutcher et al. [24]. Like MHL approach, SUMS intervention is based on existing classroom friendly pedagogical approach that can be easily delivered in the current educational systems without extracurricular or outside-of-school inputs [13]. Basically, SUMS intervention focuses on curriculum resource approaches delivered by teachers (https://mhlcurriculum.org/) where a curriculum resource guide was developed through desk review and expert inputs. The curriculum resource guide contained content and delivery methods for each SUMS intervention (MHL, MHP and MH-FA). This curriculum resource guide was adapted to the local context through key informant interviews and intervention designing workshops conducted among school teachers, students, block education officers and subject experts.

In this context, we have undertaken a cluster randomised controlled trial to evaluate the effectiveness of SUMS (Scaling Up of Mental Health in Schools) intervention (an integrated school mental health intervention) for adolescents, integrated into the classroom and delivered by teachers. We hypothesise that SUMS intervention delivered by teachers will lead to significant improvement in mental health knowledge, positive attitudes toward mental illness, and behaviours for First Aid in Mental Health as compared to ongoing regular school approach among school-going adolescent children. The ongoing regular school approach was used as comparator to reflect real-life scenario.

### Objectives

This study’s primary objective is to assess the effectiveness of evidence-informed integrated school mental health intervention (SUMS) in promoting mental health knowledge, positive attitudes toward mental illness, and behaviours for First Aid in Mental Health among adolescent school children. Furthermore, we will explore SUMS intervention’s potential influence on a range of adolescent health-related outcomes such as academic achievement, absenteeism rate, educational stress, suicidal ideation, bullying, and substance use. Additionally, we will explore the implementation process of the intervention and identify contextual factors associated with outcomes.

## Methods

### Study design

**Fig. 1:**
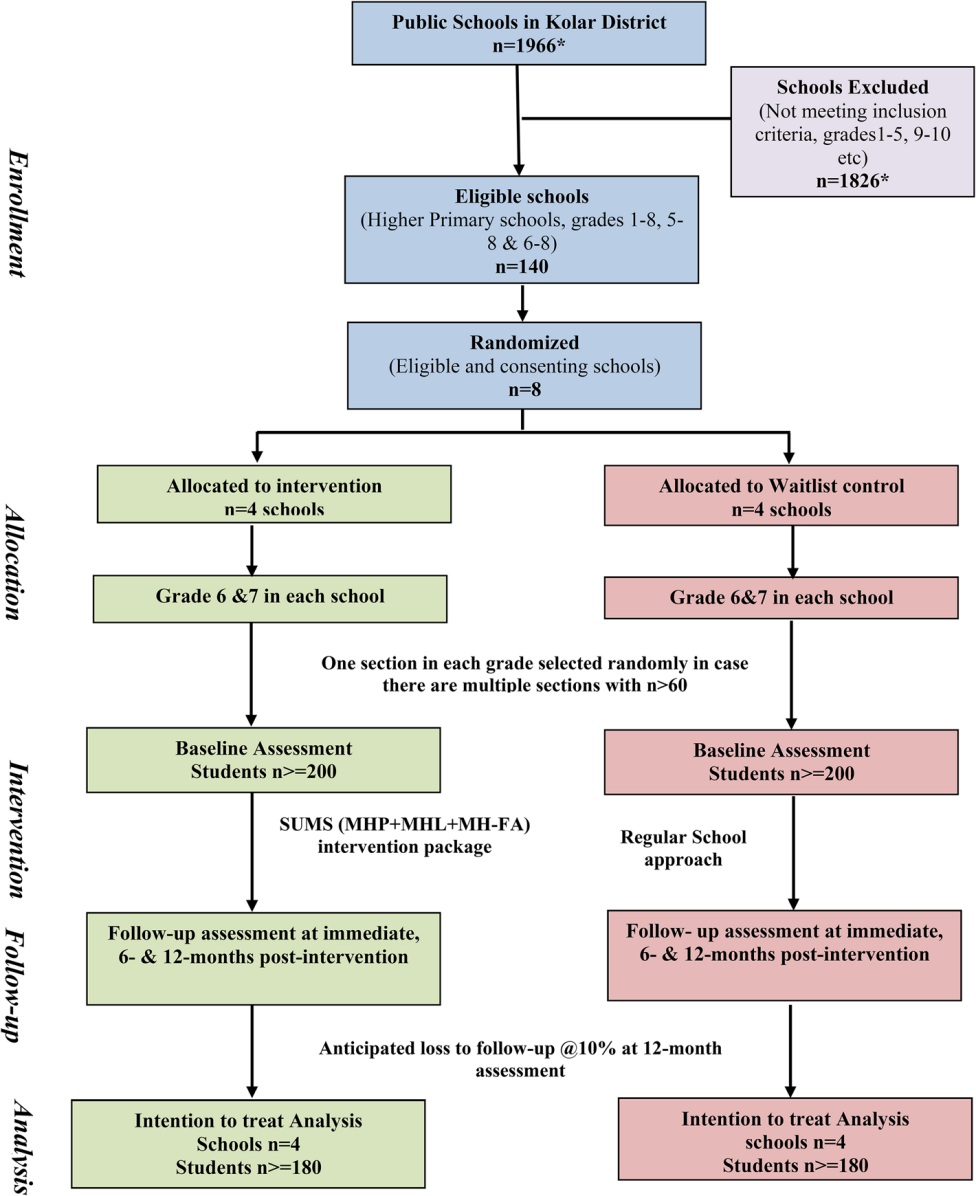
Trial Design. MHP: Mental Health Promotion. MHL: Mental Health Literacy. MH-FA: First Aid in Mental Health. * Data obtained from Department of Public Instruction Office, Kolar for the year 2016

The study will use a pragmatic, cluster randomised waitlist-controlled design with schools as unit-of-randomisation to evaluate the effectiveness of SUMS intervention. The study will follow consort guidelines for cluster randomised trial (Fig. 1).

### Study area

The study will be conducted in Srinivaspura taluka (Sub-district) of Kolar district (administrative unit of health) of Karnataka (https://kolar.nic.in/en/) in collaboration with a multi-disciplinary expert team from NIMHANS (National Institute of Mental Health And Neuro Sciences), Bangalore-India and Department of Education, Government of Karnataka, India. Kolar district is the Public Health Observatory for Department of Epidemiology, NIMHANS.

### Study setting

The Department of Education in Karnataka state has three levels of schools viz., Primary (1–5 grades), Higher-Primary (6–8 grades) and High-schools (9–10 grades). The study will be conducted in higher-primary public schools in Srinivaspura taluka of Kolar District, Karnataka. Higher-primary public schools usually include children from ages 13 to 15 years. These grades were chosen because this is the period where life skills curriculum were introduced and this period in the life span also represents the point of departure for the rapid increase in psychiatric diagnoses that occurs prior to age 25 years [24].

### Study subjects

The students from grades 6 and 7 will be included for the present study so that the students in grade-7 will have 12-month follow-up when they are in class-8.

### Inclusion criteria

All children enrolled in class 6th and 7th grade of the participating higher-primary public schools will be eligible for the present study.

### Exclusion criteria

(1) Children who did not give informed assent (2) Children whose parents/guardians did not provide written informed consent (3) Children who were absent for the baseline assessment even after two attempted visits (4) children aged> 15 years at enrolment.

### Intervention

**SUMS intervention (MHP + MHL + MH-FA)** is a classroom-based teacher-led integrated school mental health intervention that will promote positive mental health, mental health literacy and behaviours for First Aid in Mental Health among school-going adolescent school children. As described earlier, SUMS intervention takes the form of a classroom-ready resource (the curriculum resource guide) designed to be delivered by usual classroom teachers to students in grades 6 and 7 (ages 12 to 15 years). The curriculum resource guide contains 10 modules categorised under 3 units (Mental Health Promotion, Mental Health Literacy and First Aid in Mental Health). The ten modules are as follows: Positive mental health, Promoting Resilience, Promoting Mindfulness, Life Skills Education, Mental Health Literacy, Understanding Mental Health and Mental Illness, Stigma of Mental Illness, Information on specific mental illnesses, seeking help and finding support, First Aid in Mental health and strategy for mental health first Aid (Table 1). Each module’s purpose is to provide teachers with classroom-ready lesson plans, activities, and easily accessible resources. The ten modules are designed to be taught in sequence and are intended to be delivered on the usual school days through 10–15 h of classroom time.

**Table 1:** SUMS Intervention-Curriculum framework

Units	Module	Teaching method
Mental Health Promotion (MHP)	Module 1: Positive mental health Module 2: Promoting Resilience Module 3: Promoting Mindfulness Module 4: Life Skills Education	Educational session followed by Participatory and experiential activities as recommended in the resource guide (Interactive discussions, Group discussion, Co-operative play, Storytelling. Role play, Brainstorming, Debate, within-school competitions, wall-magazine, etc.,)
Mental Health Literacy (MHL)	Module 5: Understanding Mental Health and Mental Illness Module 6: Stigma of Mental Illness Module 7: Information on specific mental illnesses Module 8: Seeking help and finding support	
First Aid in Mental Health (MH-FA)	Module 9: First Aid in Mental health Module 10: Strategy for First Aid in Mental health	

### Feasibility testing for SUMS interventions

The SUMS intervention curriculum resource guide will be translated into local language Kannada and will be piloted with concerned Block Education Officers for its clarity and suitability for training school teachers and students. Perceived intervention feasibility will be assessed by adapting a 4-item Feasibility of Intervention Measure (FIM). Any suggestions by Block Education Officers will be duly accommodated to improve the feasibility [25].

### Outcomes

The following core outcomes will be used to assess the intervention. All outcome measures will be measured at the individual student level.

#### Primary outcome

The primary outcome, **Positive mental health literacy** will be measured using a 10-item Mental Health-Promoting Knowledge (MHPK-10) scale. It is a valid and reliable one-dimensional instrument measuring knowledge of factors promoting good mental health among adolescents. The MHPK-10 had demonstrated good internal validity and test-retest reliability (r = 0.74) among adolescents. For each item, respondents rate items on 1–5 likert-type scale, the extent to which they are correct or wrong. The score range is 10–50, with a higher score indicating a higher positive mental health literacy [26].

#### Secondary outcomes

##### Mental health first aid knowledge and mental health literacy

This will be measured using a 35-item univariate scale, Mental Health Literacy Scale (MHLS). The scale will be adapted for use among adolescents. The score range is 35–160, with a higher score indicating a higher mental health literacy [27].

##### Personal and public stigma towards mental health among adolescents

This will be measured using the 24-item Peer Mental Health Stigmatization Scale (PMHSS). For the present study, the 8-item personal stigma subscale will be used. Τhe scale demonstrated adequate discriminant validity, internal consistency (α = 0.75) and test-retest reliability in adolescents [28].

##### Stigmatising behavior

 will be measured using the Reported and Intended Behaviour Scale (RIBS). Scores on the RIBS range from 4 to 20, with higher scores indicating more positive attitudes. The RIBS has a test-retest reliability of 0.75, and a Cronbach’s α of 0.86 [29].

##### Mental health help-seeking

This will be assessed using a 24-item Inventory of Attitudes towards Seeking Mental Health Services (IASMHS) scale. It consists of three subscales; Psychological Openness, Help-seeking Propensity, and Indifference to Stigma. The total help-seeking score is calculated as a sum of the subscale totals with higher scores indicate more favourable attitudes towards help-seeking [30].

##### Psychological distress

A 6-item self-administered **Kessler psychological distress scale (K6)** with good internal consistency (Cronbach’s alpha = 0.84) will be used. The total score ranges from 0 to 24 with a higher score indicating higher distress [31].

##### Resilience

A 25-item Connor-Davidson Resilience Scale (CD-RISC) will be used. The CD-RISC is a valuable measure to assess resilience among adolescents in low-income settings. Psychometric properties of the Kannada version of CD-RISC were established in a sample of adolescent girls from low-income settings. The total score ranges from 0 to 100, with higher scores reflecting greater resilience [32].

##### A semi-structured interview schedule

 will measure socio-demographic details and contain case vignettes/questions to assess MH-FA knowledge and intentions to provide MH-FA (proxy measures of behaviour change).

##### Exploratory outcomes

A compelling body of evidence points to the potential influence of SUMS intervention on a range of adolescent health-related outcomes such as academic achievement, absenteeism rate, educational stress, suicidal ideation, bullying and substance use, which will be explored.

##### Pre-testing of study instruments

The pre-validated study instruments will be translated into local language and back-translated for conceptual equivalence. For pre-testing, a sample size of 42 is estimated to establish reliability of at least 0.85, against acceptable value of 0.7 (with α = 0.05; β = 0.20) for a minimum of 6 items [33]. The translated study instruments will be pre-tested on a representative sample for cultural relevance and comprehensibility and validated for internal consistency before their final inclusion. The final study instrument administered will be bilingual.

##### Sample size and power

Positive mental health literacy measured using the 10-item Mental Health-Promoting Knowledge (MHPK-10) scale is considered primary outcome for the present study [26]. However, the Intra-Cluster Correlation for MHPK-10 score is unknown. Based on evidence from similar adolescents studies, we assumed a conservative and modest estimate of ICC (ρ) between 0.01 and 0.05 [34, 35]. From the Kolar District Report (as on 30/3/2016), an estimated average of 50 students is expected from grade-6 and 7 of each higher-primary schools. With a need for 4 schools per arm with 50 students per school, we powered the trial to be able to detect a standardised difference (difference in means/SD) of 0.37–0.56 (medium effect size) for MHPK-10 scores between intervention and control groups for a two-sided level of significance at 5% (*α* = 0.05) with 85% power. For the above assumption, the design effect varied from 1.5 to 3.5 for ICC (ρ) values from 0.01 to 0.05, respectively [36]. We expect a minimum sample size of 400 across 8 schools for 85% power. Even with a 5% loss to follow-up, the study will still have more than 80% power.

##### Randomisation

From the eligible higher-primary school-list obtained from the education department of Srinivaspura taluka, eight schools will be *randomly selected* using computer-generated random-number by an independent researcher. The selected schools will then be assigned to the intervention, and waitlist-control condition (receiving intervention after the post-assessment in intervention-group) by the independent researcher through the *random-allocation* sequence. To ensure allocation concealment, randomly selected schools will be assigned unique study numbers that will not be known to the independent researcher at the central office. The sequence will be delivered through sealed, opaque envelopes.

The selected-schools will be invited to participate in the study, and briefing-meetings will be conducted to inform principals of selected-schools regarding the study. Information on any initiatives/activities related to target outcomes in the selected-schools will be obtained. Local consortium and local stakeholders for education will be interacted to ensure minimal refusal of the selected schools. In case of refusal, the refusing school will be replaced with another school from the eligible school-list. A due care will be taken to prevent possible contamination so that no control-schools will be selected within 5 km radius of intervention-schools. An alternate covariate-constrained randomisation will be considered only if we do not reach an acceptable baseline balance through simple-randomisation.

##### Blinding

Given the nature and complexity of intervention & its delivery, it will not be possible to completely blind or conceal the intervention from the teachers, students, and field-staff. The trial will be open-label. Nonetheless, the study-statistician will be blinded till the database is locked for final-analysis.

##### Student-recruitment

Enrolment will start after obtaining assent and parental-consent from the eligible students. In the case of multiple sections with > 60 students in each grade, one section will be randomly selected from each grade. The intervention will be provided to all students in the selected grades irrespective of their number. The teachers will be free to provide intervention for other sections, but data will not be collected.

##### Intervention-schools

For the schools randomised to the intervention arm, the teachers nominated by their designated superior officials will be trained on the curriculum resource guide content in a 3-day workshop. The teachers who provide consent to participate in the research, complete the training, stay in the selected-schools for the study period and deliver the intervention will be selected. Following baseline assessment of participating students, the trained teachers will then proceed with the implementation of SUMS Intervention Curriculum Resource Guide, which requires approximately 10–15 h of classroom time over two months. In the intervention delivery, the teachers will be given pedagogical flexibility but will be instructed to adhere to the standardised core content, recommended lesson plans and procedures to maintain fidelity (Table 2). Booster sessions will be provided at 2-months and 5-months following the initial intervention.

**Table 2:** Quality assurance and data management

**Field-team:** The field-team will comprise of Project-Assistant who will be trained in standard protocols of questionnaire administration for data collection, coordinate with local stakeholders, monitor compliance of intervention protocol and its deviations and also conduct public-engagement activities.	
**Post-recruitment retention strategies:** For the Project-Assistant, all the activities required to achieve the trial goals will be communicated clearly. A weekly meeting will be held to sort out the field related issues. In addition, the teachers, principals, and other key stakeholders will be informed on the trial’s status and progress through newsletters.	
**Trial-Monitoring:** The Project-Assistant will check for intervention-delivery, protocol-adherence, data-collection and reporting using supervisory check-list during their monthly visits. Principal-Investigator will review this monthly visit reports during monthly review meetings. The central-research team (comprising of Principal-Investigator and other expert-collaborators) will have meetings once in 6 months at NIMHANS to review the progress, work plan, discuss and troubleshoot any unresolved field issues. A register will be maintained for all review meetings. Constant support will be obtained from the expert collaborators through tele-meetings. A Trial Steering Committee (TSC) and Data Safety Monitoring Board (DSMB) will be constituted independently of the research team, the members of whom will have no conflict of interest. The TSC will comprise experienced experts to oversee the trial and independently evaluate the trial to monitor progress, intervention delivery, protocol adherence and data management. A three-member DSMB will monitor the data as the trial progresses to identify any emerging safety concerns for the study participants and problems in the study’s conduct. DSMB will decide on the continuation of trial following interim analysis.	
**Intervention Fidelity:** Teachers will be encouraged to fill a protocol-adherence checklist. The educational-intervention delivered by the teacher will be audio-recorded to assess fidelity of intervention delivery. A random sample of audiotaped sessions will be evaluated for each teacher using fidelity checklists for intervention-delivery based on the standardised core content of the SUMS intervention curriculum resource guide. Feedback will be provided to support the booster-training sessions. Our fidelity assessment will also incorporate feedback and recommendations of the independent evaluation and reports of supervisory-checklist.	
**Data Management:** The study will have meaningful engagement with key stakeholders across all study phases. All data stored securely in-line with our data-management protocol will protect the confidentiality of participants. Any personal identifiers of study participants will be removed before analysis. Audiotapes and transcripts containing participants identity (if any) will be destroyed post-analysis of data. Participant’s identity will not be revealed in any of the publications related to this research. The principal investigator will have access to the final trial dataset.	

##### Control-schools

Control-schools will continue to provide regular teaching as usual of the existing course to their students. The control group will receive the interventions at the end of the 12-month follow-up assessment in intervention-schools. The teachers in the waitlist control arm will be trained just one month before their scheduled intervention to prevent contamination.

##### Parental-consent

An information letter detailing the intervention program and data collection along with consent-form will be sent to the parents/guardians of participating class for parental-consent. Parental-consent will be recorded with a signature/thumbprint. Existing school communication channels (letter, diary or parent’s teachers meeting) will be used to maximise the parental-consent for child participation. Parents will be provided an opportunity to contact the field-team to discuss the study and their participation. An attempt will be made to meet the non-responding parents to obtain their consent. Children without parental consent in the selected class will be permitted to attend intervention sessions, but data will not be collected from them.

##### Discontinuation-criteria

The students may withdraw their participation at any point in time during the study. An attempt will be made to document the reasons for withdrawal.

##### Timing of outcome-measurement

**Fig. 2:**
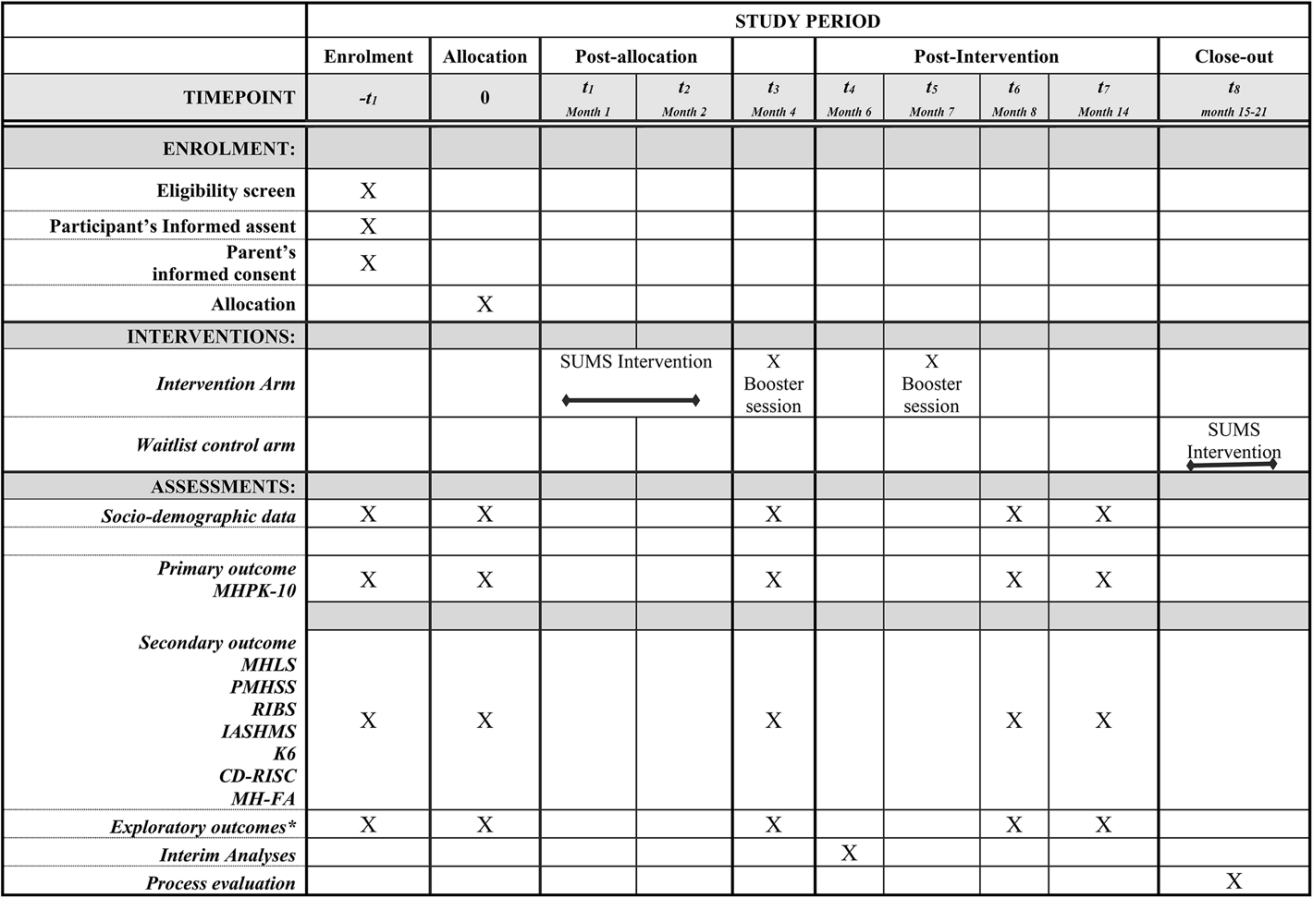
Trial process. MHPK: Mental Health Promoting Knowledge; MHLS: Mental Health Literacy Scale; PMHSS: Peer Mental Health Stigmatization Scale; RIBS: Reported and Intended Behaviour Scale; IASMHS: Inventory of Attitudes towards Seeking Mental Health Services; K6: Kessler psychological distress scale; CD-RISC: Connor-Davidson Resilience Scale; MH-FA: First Aid in Mental Health. *Exploratory outcomes include academic achievement, absenteeism, educational stress, suicidality, bullying and substance use

Data will be collected using self-administered questionnaire. Students will complete the survey during class time in the presence of trained project assistant/ teacher to assist the children with reading, writing and answering any queries. Data will be collected at 4-timepoints from both intervention and control schools: baseline and 6-weeks, 6-months and 12-months post-intervention (Fig. 2). To maximise data completeness, all effort will be made to obtain data from absentee students through liaison with key school-staff.

##### Adverse-events

We do not anticipate any risk to the participating students due to our interventions. However, potential discomfort due to sensitive questions of survey assessment related to self-perception and well-being cannot be ruled out when answering in the classroom. Due-care will be taken to ensure the privacy and confidentiality of students while filling the survey forms. A register & form will be maintained to record unexpected severe events during the trial. Any students who require professional mental help during the study will be referred to the district mental health team for further management.

##### Process evaluation

The process evaluation will include concurrent fidelity assessment and a qualitative study (focus group/in-depth interviews) among students/teachers during the final stages of the trial to explore the implementation of the intervention and identify contextual factors associated with outcomes. Teachers will be provided with questionnaires to evaluate the Curriculum Guide and to provide feedback on their experience.

#### Plan of analysis

The study tools/measures will be examined for internal consistency. Demographic and baseline characteristics will be summarised using descriptive statistics. Principal-analyses will be intention-to-treat which will take into account clustering by the school and repeated measures. To assess the effectiveness of interventions, outcomes will be compared between two trial arms using linear-mixed-model analysis for continuous outcomes and generalised linear mixed models or generalised estimating evaluation (GEE) for binary outcomes. The analysis will include all participants with at least one outcome measurement at 6 weeks post-intervention. A per-protocol analysis will also be conducted along with intention-to-treat analysis to examine the influence of missing data patterns. Multiple imputation will be attempted for missing values. The analysis will be adjusted for potential confounders that are identified a-priori during the study. Subgroup analysis will be performed for important socio-demographic variables. Statistical significance will be set at *p*-value< 0.05, and Bonferroni’s correction will be made for multiple testing (if any).

For qualitative analysis, all the verbatims of interviews/group discussions will be transcribed and translated into English for thematic analysis.

##### Interim-analyses

Interim analyses is planned for the follow-up data collected at 6-weeks post-intervention and will be submitted to Data Safety & Monitoring Board (DSMB) for further review (Table 2).

##### Stopping-rule

The DSMB will be asked to advise stopping the trial if they have proof beyond doubt that intervention is futile. The trial’s stopping-rule will be based on evidence-of-futility in > 70% of outcome measures (both primary &secondary) at interim-analyses and critical feedback from the local stakeholders.

#### Trial status and review

The trial was reviewed and funded by Indian Council of Medical Research (Id: 2017–4697 version F1 dated 02 September 2019). The trial was reviewed and approved by the Institute Ethics Committee, NIMHANS (No.NIMHANS/19^th^IEC (BS&NS DIV.)/2019 on 2019-07-17. The trial was registered at Clinical Trials Registry-India with identification code: CTRI/2019/07/020394 dated 2019-07-29. Due to COVID-19 Pandemic and school closures, recruitment for the trial is anticipated after March 2022.

## Discussion

This pragmatic, cluster randomised waitlist-controlled design will examine the effectiveness of classroom-based teacher-led integrated school mental health intervention in improving school-going adolescent children’s mental health and well-being. To the best of our knowledge, this is the first study to integrate MHL with MHP and MH-FA for promoting mental health well-being among adolescent school children in India.

The trial is an extension of a series of focused research activities undertaken in Child and Adolescent Mental Health (CAMH) in Kolar district, Karnataka. The activities included situational analysis, numerous consultation workshops with teachers and various training programmes organised in-collaboration with the education department in Kolar. Following interactive discussions with NIMHANS experts and a national-level symposium, a road-map and draft action plan for integrated CAMH services in a district was finalised. The present trial is a part and extension of the action plan developed for promoting CAMH. Though the mental health intervention domains (MHP+ MHL + MH-FA) are closely interlinked with considerable cross-cutting areas of action, we intentionally compartmentalised it for better focus and delivery. With a need to build a more substantial evidence base on effective School Mental Health Promotion approaches in low-income countries, the study builds on the facilitating role of school climate in promoting mental health and related outcomes among adolescents and evaluates its effectiveness through a robust and systematic methodology.

### Limitations

As cultural aspects are likely to influence the effectiveness of SUMS interventions, the trial results should be interpreted within the given cultural context. As this study is being conducted in a relatively small geographical area, a larger multi-centre trial is required to better understand SUMS interventions’ replicability and scalability, especially in different cultural settings.

India has recently launched the Health and Wellness Ambassador initiative under the School Health Programme of Ayushman Bharat Mission [37]. This initiative trains two identified teachers from each school to facilitate the healthy survival of school children under 11 themes, including emotional well-being and mental health. In this context, the evidence obtained from this study will have implications for implementing and operationalising the Health and Wellness Ambassador initiative in schools. Further, the barriers and challenges identified in the present study will provide valuable inputs to improve school climate for promoting mental health among school-going adolescents, especially in resource-poor settings. This study will contribute significantly towards transforming the vision of shifting from “survival to healthy survival” of adolescent children in developing countries into reality.

## Data Availability

Data sharing is not applicable to this article as no datasets have yet been generated or analyzed.

## Abbreviations

CAMH: Child and Adolescent Mental Health
CD-RISC: Connor-Davidson Resilience Scale
DSMB: Data Safety & Monitoring Board
HPS: Health Promoting Schools
IASMHS: Inventory of Attitudes towards Seeking Mental Health Services
KII: Key informant interview
MH-FA: First Aid in Mental Health
MHL: Mental Health Literacy
MHLS: Mental Health Literacy Scale
MHP: Mental Health Promotion
MHPK: Mental Health Promoting Knowledge
NIMHANS: National Institute of Mental Health And Neuro Sciences
PMHSS: Peer Mental Health Stigmatization Scale
RE-AIM: Reach, Effectiveness, Adoption, Implementation, and Maintenance
RIBS: Reported and Intended Behaviour Scale
SUMS: Scaling Up of Mental health in Schools
TSC: Trial Steering Committee
